# Gait in depression: a bibliometric analysis and knowledge mapping of research trends over the past 20 years

**DOI:** 10.3389/fpsyt.2025.1457176

**Published:** 2025-08-20

**Authors:** Shao-Kui Kan, Nuan-Nuan Chen, Bo Peng, Ying-Li Zhang

**Affiliations:** ^1^ Depressive Disorders Ward I, Shenzhen Kangning Hospital/Shenzhen Mental Health Center/Shenzhen Clinical Research Center for Mental Disorders, Shenzhen, China; ^2^ Geriatric Psychiatry Cognitive Disorder Ward, Shenzhen Kangning Hospital/Shenzhen Mental Health Center/Shenzhen Clinical Research Center for Mental Disorders, Shenzhen, China

**Keywords:** depression, gait, bibliometric, VOSviewer, CiteSpace, visual analysis

## Abstract

**Background:**

Depression carries a high risk of suicide, with many individuals failing to receive treatment due to diagnostic challenges and stigma. Gait is linked to depression, underscoring the importance of gait analysis in the diagnosis and treatment of depression. However, comprehensive and objective evaluations of the current research on gait in depression are lacking. This study aims to use bibliometric analysis and knowledge mapping to clarify research trends and status.

**Objective:**

This study employs bibliometric analysis to investigate gait in depression, summarizing historical and current trends while predicting future directions. This analysis will aid researchers and policymakers in understanding evolving trends and prioritizing research resources effectively.

**Methods:**

We conducted a computer-based search of the Web of Science core collection to identify articles and reviews related to depression and gait. Bibliometric analysis, involving the analysis of aspects such as countries or regions, institutions, authors, journals, keywords, and references, was performed using Excel 365, CiteSpace, and VOSViewer.

**Results:**

The analysis included a total of 848 publications from 2005 to 2024. The results showed a phased increase in publications, peaking in 2020 with 102 publications, followed by a gradual decline. Citations in this field showed a yearly increase, peaking in 2022 with 3920 citations before subsequently declining. The United States was identified as the most productive and influential country in this field, with the highest number of publications and citations. They have the institutions with the highest publications and citations. Leading authors in this field include Verghese Joe, Shimada Hiroyuki; and Rochester Lynn. Key journals include BMC Geriatrics, Journals of Gerontology Series and Medical Sciences, and Journal of the American Geriatrics Society. Frequently mentioned keywords in this field are depression, gait, gait speed, older adults, and dementia. Identification of distinctive gait patterns in depression, gait characteristics in the elderly, association between gait and cognitive decline, and interventions for abnormal gait constitute current research forefronts in this domain.

**Conclusions:**

This study is the first to utilize bibliometrics to visualize research in the field of gait-related depression. It reveals research trends and frontiers, providing valuable references for scholars seeking important research topics and potential collaborators.

## 1 Introduction

Depression encompasses enduring feelings of low mood and reduced interest in activities, profoundly interpersonal relationships as well as academic and occupational performance. An estimated 3.8% of the global populace grapples with depression, with this condition affecting 5% of the adult population (comprising 4% of men and 6% of women) (1). Among those aged 60 and above, the prevalence rises to 5.7% (1). Notably, depression occurs approximately 50% more frequently in women compared to men. Globally, over 10% of women experience depression during pregnancy or shortly after giving birth (2).

Depression is associated with decreased life satisfaction, impaired psychosocial functioning, and heightened rates of disability, even leading to suicide. Effective treatments for mental disorders exist; however, more than three-quarters of people in low- and middle-income countries do not receive any form of treatment (3). Furthermore, over half of individuals with depression have not received treatment, often due to challenges in diagnosis (4, 5). Another persistent issue is the use of traditional questionnaire-based approaches for depression, which can increase the risk of misdiagnosis and subsequent inappropriate treatment in primary care settings (6–8). From the patient’s perspective, internalized stigma associated with depression correlates with an intensified emphasis on each identified barrier to treatment, including perceived need, negative treatment expectations, and structural obstacles (9). Furthermore, individuals with depression encounter stigma related to antidepressants, along with significant challenges in accessing antidepressant treatment and in doctor-patient communication (10). Evidence suggests that motor symptoms, including gait abnormalities, are crucial manifestations of depression (11, 12). Specifically, gait and postural control are governed by a complex neural network that is also implicated in the pathophysiology of major depression (13). In their 2012 longitudinal study spanning 16 years among the elderly, Joost B. Sanders and colleagues (14) found that walking speed predicted the onset of depression. Additionally, in subsequent research (15), it has been observed that gait correlates with the severity of depression, achieving an accuracy rate of 84.91% in predicting depression through gait analysis. Recently, Yameng Wang et al. (16) have discovered that individuals suffering from depression demonstrate a distinct gait in contrast to participants in the control group. This is marked by a decelerated walking pace and diminished movements across various parameters, including reduced left-arm swing, right-arm swing, head posture, right stride length, left toe clearance, and right toe clearance.

Bibliometrics is a methodological approach that utilizes qualitative and quantitative methods to analyze, evaluate, and manage literature and information resources (17). It is widely applied across various disciplines (18–20). It integrates techniques from information science, statistics, and computer science, primarily aiming to measure and assess the output, impact, and trends of scientific research (17). Bibliometrics involves analyzing indicators such as citation analysis, author collaboration networks, international collaboration networks, institutional collaboration networks, journal citation analyses, and trends in research topics. It aids researchers and decision-makers in understanding the dynamic changes within academic fields, supporting the formulation of research policies and allocation of resources. Despite the rapid evolution of research into depression-related gait characteristics over the past two decades, there exists a deficiency in contemporary bibliometric analyses. Hence, this study endeavors to undertake a bibliometric analysis of depression-related gait research using VOSviewer and CiteSpace, bibliometric software tools. The objective is to discern trends and cutting-edge focal points over the last twenty years, thereby assisting researchers and policymakers in comprehending the dynamic shifts within the scholarly domain and facilitating informed decisions on research policy and resource allocation.

## 2 Materials and methods

### 2.1 Search strategy and data retrieval

The bibliometric analysis in this study is based on data from the Web of Science Core Collection (WOSCC), a widely used academic database (21). Our retrieval methodology employs “TS= (“Depression” OR “Emotional Depression” OR “Depression, Emotional” OR “Depressive Disorder” OR “Depressive Disorders” OR “Disorder, Depressive” OR “Disorders, Depressive” OR “Neurosis, Depressive” OR “Depressive Neuroses” OR “Depressive Neurosis” OR “Neuroses, Depressive” OR “Depression, Endogenous” OR “Depressions, Endogenous” OR “Endogenous Depression” OR “Endogenous Depressions” OR “Melancholia” OR “Melancholias” OR “Unipolar Depression” OR “Depression, Unipolar” OR “Depressions, Unipolar” OR “Unipolar Depressions” OR “Depressive Syndrome” OR “Depressive Syndromes” OR “Syndrome, Depressive” OR “Syndromes, Depressive” OR “Depression, Neurotic” OR “Depressions, Neurotic” OR “Neurotic Depression” OR “Neurotic Depressions”)” AND TS= (“Gait” OR “Gaits”). The most recent search update was performed on June 21, 2024. The analysis encompasses publications spanning from January 1, 2005, to June 21, 2024. Included are 848 documents, restricted to types “article” and “review,” and filtered for English language. These documents were exported in plain text format for further examination.

### 2.2 Statistical analysis and visualization of data

We employed CiteSpace 6.3.R3 Advanced, developed by Chaomei Chen (22), and VOSviewer, created by Nees Jan van Eck et al. (23), to conduct comprehensive visual analyses. By extracting critical information such as titles, authors, affiliations, countries or regions, publication journals, keywords, and references, we visualized collaborative networks among countries, institutions, and authors, keyword co-occurrence networks, keyword timelines, author and journal co-citation analyses, and reference co-citation analysis. Additionally, Microsoft 365 Excel was utilized for data aggregation, organization, and visualization. As all data utilized in this study were sourced from public databases, ethical review was not required.

## 3 Results

### 3.1 Retrieval diagram, annual publications, and citations

**Figure 1:**
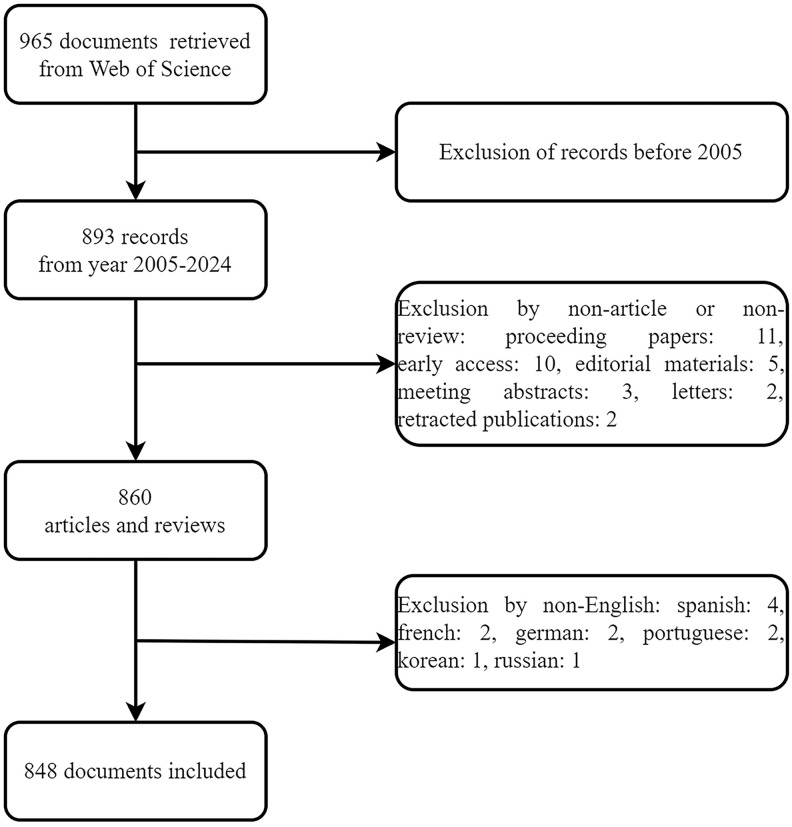
Flow diagram.

**Figure 2:**
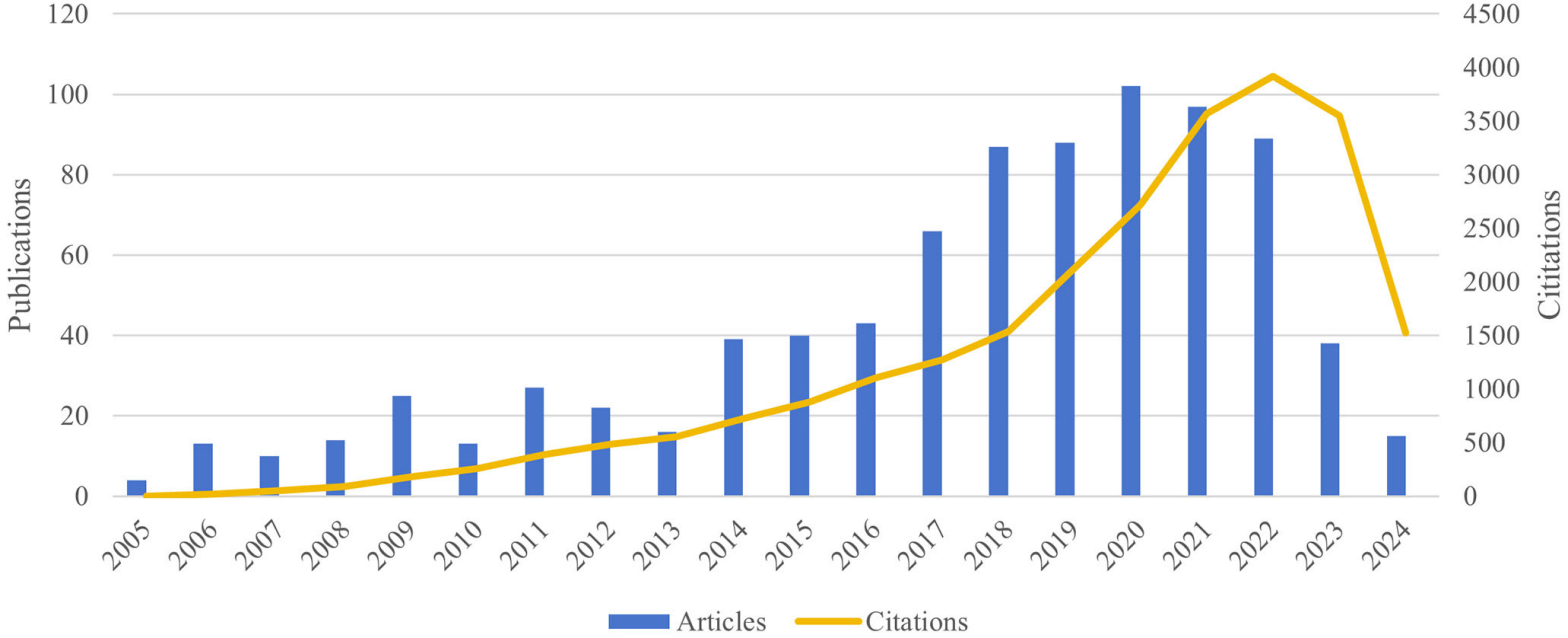
Annual publication output and cumulative citation impact, 2005–2024.

We retrieved 965 records from searches spanning 2005 to 2024, with English language and selections limited to articles and reviews. Ultimately, 848 documents were included in the analysis (**Figure 1**). **Figure 2** illustrates the annual publication trends and citation frequencies of gait-related depression articles from 2005 to 2024. The annual publication count demonstrates a fluctuating upward trend, reaching its peak of 102 publications in 2020, followed by a gradual decline. Conversely, the citation frequency of gait-related depression articles shows a steady annual increase, reaching its apex of 3920 citations in 2022 before declining in subsequent years.

### 3.2 Distribution of countries or regions

There are a total of 59 countries or regions involved in research on gait-related depression. **Table 1** delineates the top 10 countries or regions by publication volume, citation frequency, and the highest average citations per article. The United States (USA) leads in publication volume with 302 articles, while other countries or regions have fewer than 100 publications each. Similarly, the USA tops the citation frequency with 10,683 citations, followed by the United Kingdom with 3,445, Canada with 2,676, and Australia with 2,374 citations; citation frequencies for other countries or regions are below 2,000. Furthermore, Vietnam ranks highest in average citations per article at 76.00, followed closely by Finland at 53.50. All other countries or regions have average citations per article below 50.

**Table 1:** Top 10 countries or regions by publication count and citation frequency.

Rank	Countries/Regions	Publications	Country/Regions	Citations	Country/Regions	Citations per article
1	USA	302	USA	10683	Vietnam	76.00
2	Canada	76	United Kingdom	3445	Finland	53.50
3	United Kingdom	75	Canada	2676	Austria	49.89
4	China	70	Australia	2374	Israel	46.53
5	Germany	56	Germany	1761	Argentina	46.50
6	Italy	56	Italy	1617	Chile	46.00
7	Japan	56	Netherlands	1609	United Kingdom	45.93
8	Australia	55	Japan	1317	Netherlands	43.49
9	Brazil	41	China	1274	Australia	43.16
10	Spain	38	Brazil	1201	Malaysia	38.83

**Figure 3:**
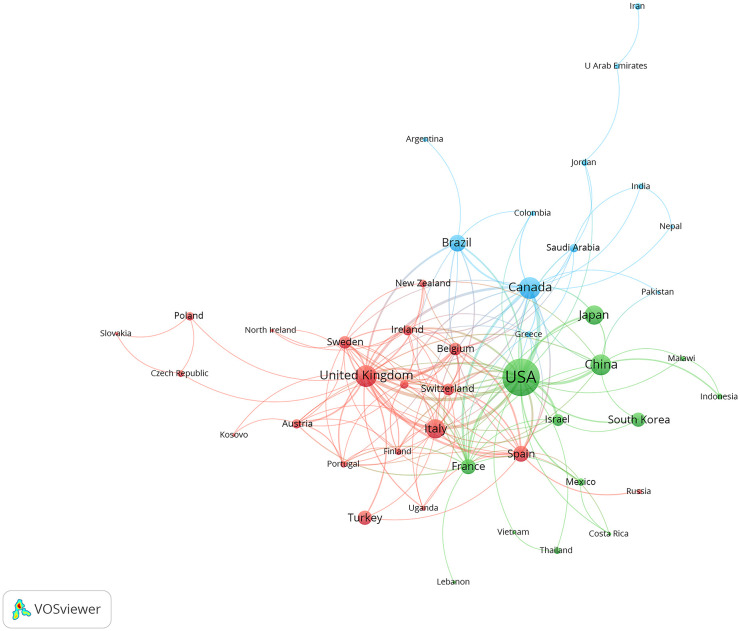
International and regional collaboration network.

**Figure 3** depicts the collaboration dynamics among countries and regions engaged in gait-related depression research. Utilizing VOSviewer, these entities are clustered based on the intensity of their collaboration, distinguished by varying colors. The green cluster comprises countries such as USA, China, and Japan; the blue cluster includes Canada, Brazil, and Greece; while the red cluster features the United Kingdom, Italy, and Belgium. Node sizes reflect publication volumes, highlighting larger nodes for countries like USA, United Kingdom, and Canada. The thickness of connecting lines indicates the strength of inter-country collaborations. Results underscore robust ties between the USA and countries such as China, Japan, and Canada, underscoring their pivotal roles in the domain of gait-related depression research.

### 3.3 Distribution of institutions

There are 1516 affiliations contributing to research on gait-related depression. **Table 2** details the top 10 affiliations by publication count and citation frequency. Leading in publication count is the University of Pittsburgh with 29 articles, followed by the University of California, San Francisco with 21 articles; all other affiliations have fewer than 20 publications. The University of Pittsburgh also tops citation frequency with 1204 citations, while other affiliations have citation frequencies below 1000.

**Table 2:** The top 10 institutions by publication volume and citation frequency.

Rank	Institutions	Publications	Institutions	Citations
1	University of Pittsburgh (USA)	29	University of Pittsburgh (USA)	1204
2	University of California, San Francisco (USA)	21	University of California, San Francisco (USA)	902
3	Harvard Medical School (USA)	20	University of Washington (USA)	832
4	National Center for Geriatrics and Gerontology (Japan)	20	Harvard University (USA)	806
5	Columbia University (USA)	19	Newcastle University (United Kingdom)	774
6	Albert Einstein College of Medicine (USA)	18	National Center for Geriatrics and Gerontology (Japan)	728
7	Duke University (USA)	17	Harvard Medical School (USA)	712
8	National Institute on Aging (USA)	15	National Institute on Aging (USA)	709
9	University of British Columbia (Canada)	15	Tel Aviv University (Israel)	669
10	University of Maryland (USA)	15	Queen’s University (Canada)	578

**Figure 4:**
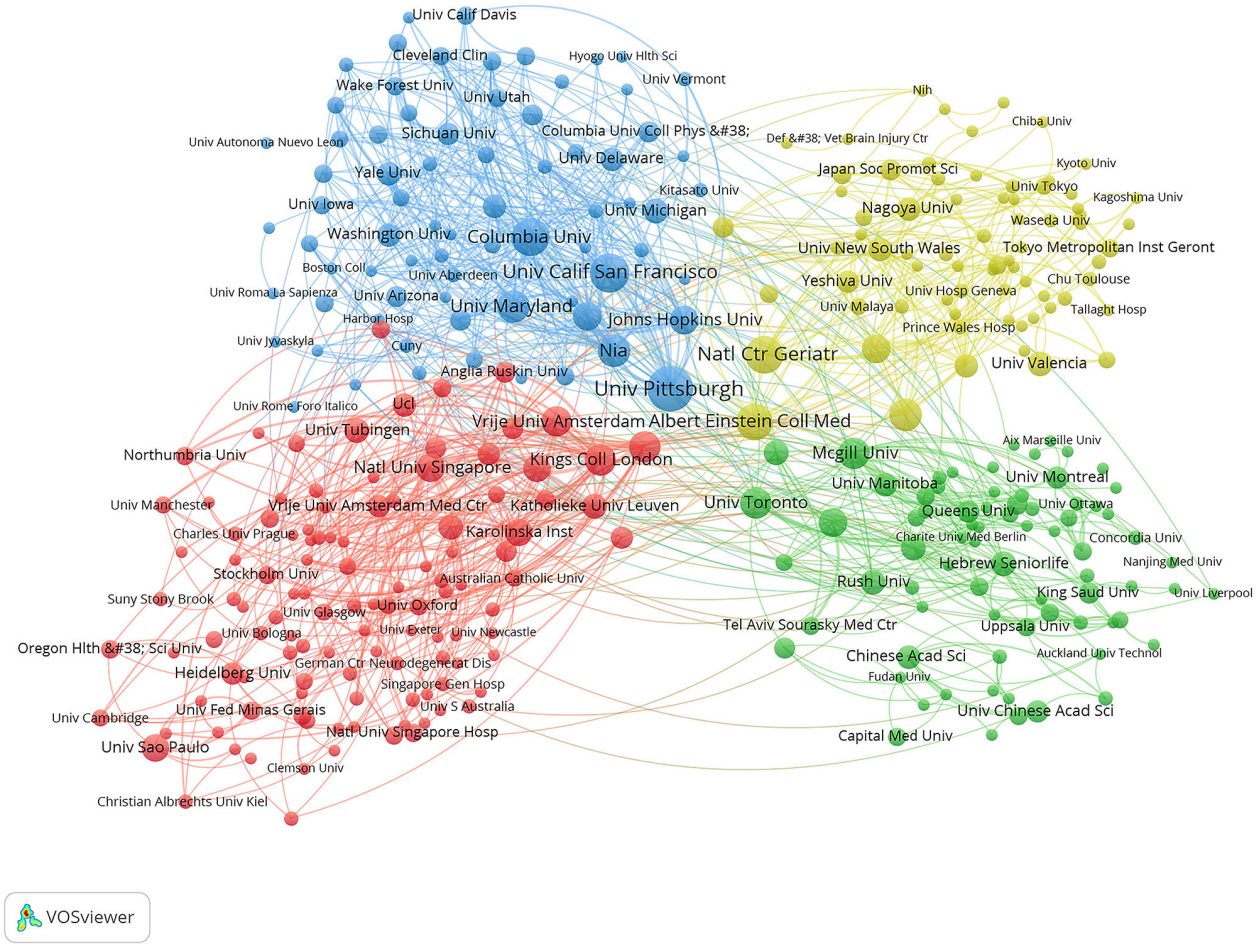
Institutional collaboration network.

**Figure 4** visualizes the collaborative network among institutions engaged in research on gait-related depression. Using VOSviewer, these institutions are categorized into 4 distinct clusters based on the intensity of their collaboration, indicated by different colors. The red cluster includes institutions such as King’s College London, Katholieke Universiteit Leuven, and Newcastle University; the green cluster comprises McGill University, Hebrew SeniorLife, and University of Toronto; the blue cluster features University of Pittsburgh, University of California, San Francisco, and Columbia University; and the yellow cluster includes University of British Columbia, Albert Einstein College of Medicine, and University of Western Ontario. These institutions also represent the nodes with the strongest collaborative ties within their respective clusters. Node sizes reflect the publication volumes, highlighting larger nodes for institutions like University of Pittsburgh, University of California, San Francisco, Harvard Medical School, University of British Columbia, and University of Toronto, which have substantial contributions in this research domain.

### 3.4 Distribution of authors

By examining the authors with the highest number of publications and co-citation frequencies in gait-related depression research, one can discern the research prowess of these authors as well as the prevailing research trends in this domain. A total of 4,781 authors have engaged in studies on gait-related depression. **Table 3** delineates the top 10 authors ranked by publication volume and citation frequency. Verghese, Joe leads with 12 publications, followed by Shimada, Hiroyuki with 11 publications, and Rochester, Lynn with 10 publications, while all other authors have fewer than 10 publications. In terms of citation frequency, Shimada, Hiroyuki ranks highest with 546 citations, followed by Doi, Takehiko with 516 citations, and Tsutsumimoto, Kota with 516 citations; all other authors have fewer than 500 citations.

**Table 3:** The top 10 authors by publication volume and citation frequency.

Rank	Author	Publications	Author	Citations
1	Verghese, Joe	12	Shimada, Hiroyuki	546
2	Shimada, Hiroyuki	11	Doi, Takehiko	516
3	Rochester, Lynn	10	Tsutsumimoto, Kota	516
4	Brown, Patrick J.	9	Makizako, Hyuma	497
5	Lord, Stephen R.	9	Michalak, Johannes	486
6	Makizako, Hyuma	9	Suzuki, Takao	480
7	Roose, Steven P.	9	Herman, Talia	478
8	Rutherford, Bret R.	9	Verghese, Joe	471
9	Doi, Takehiko	8	Hausdorff, Jeffrey M.	444
10	Tsutsumimoto, Kota	8	Rochester, Lynn	438

**Figure 5:**
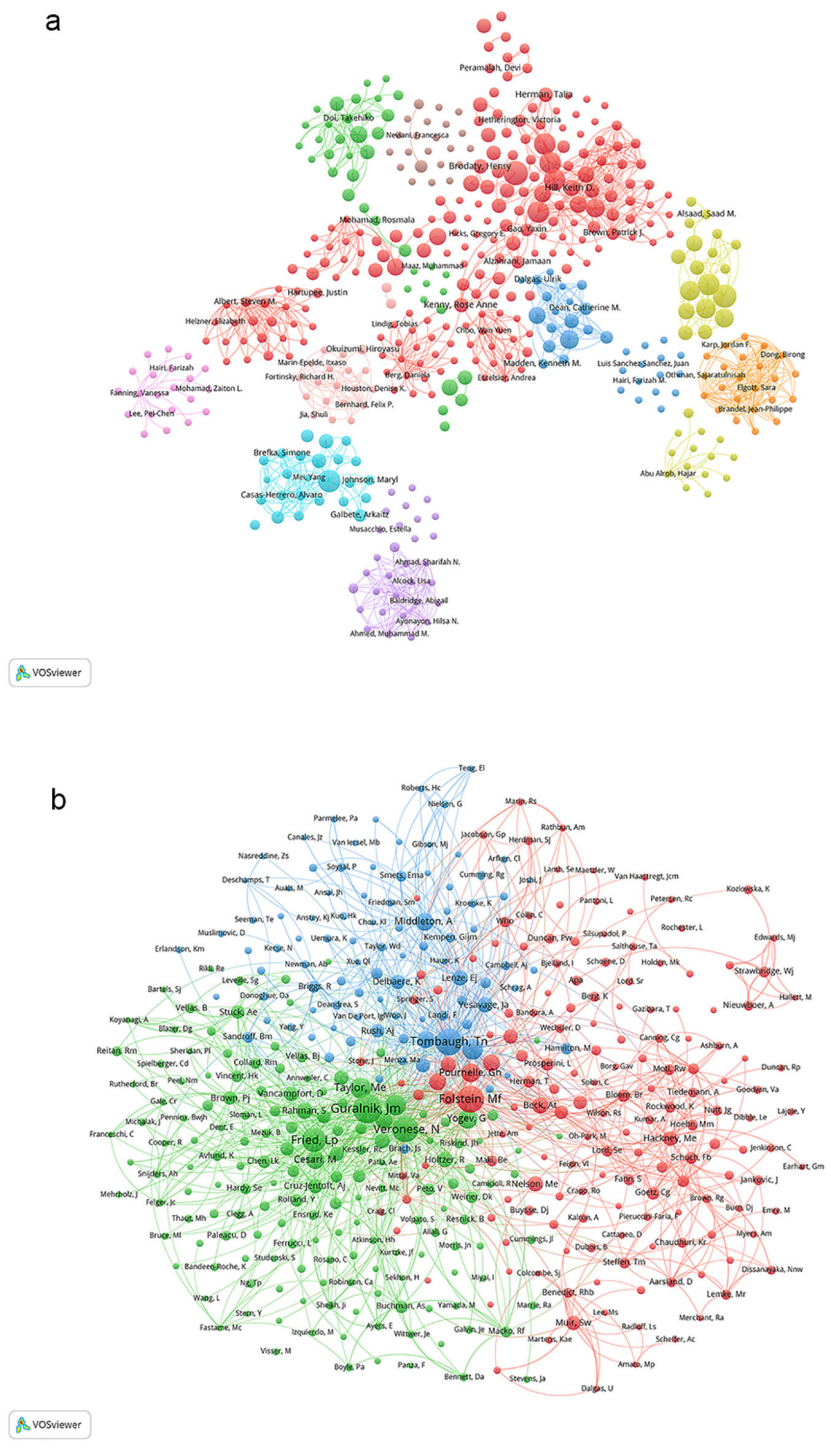
**(A)** Author collaboration network. **(B)** Co-citation author network.

**Figure 5a** delineates the collaborative network among authors engaged in gait-related depression research. Leveraging VOSviewer, authors are clustered into 10 distinct groups based on the intensity of their collaborations, each represented by a different color. The red cluster encompasses authors such as Hill, Keith D, Brodaty, Henry, and Albert, Steven M; the green cluster includes Doi, Takehiko, Abe, Takafumi, and Akishita, Masahiro; the yellow cluster consists of Abu Alrob, Hajar, Alsaad, Saad M, and Richardson, Julie; the light purple cluster comprises Fanning, Vanessa, Hairi, Farizah, and Mohamad, Zaliton L; the light blue cluster features Brefka, Simone, Casas-Herrero, Alvaro, and Johnson, Mary L; the purple cluster includes Ahmad, Sharifah N, Baldridge, Abigail, and Ayonayon, Hilsa N; the light pink cluster encompasses Houston, Denise K, Jia, Shuli, and Bernhard, Felix P; the blue cluster includes Dean, Catherine M., Dalgas, Ulrik, and Kenneth M; the orange cluster comprises Dong, Birong, Karp, Jordan F., and Elgott, Sara; and the brown cluster includes Neviani, Francesca, Stubbs, Brendon, and Herman, Talia. Node sizes are indicative of the publication volumes of the authors, while the thickness of the connecting lines signifies the strength of the collaborative ties among them. Authors with the highest publication volumes include Verghese, Joe; Shimada, Hiroyuki; and Rochester, Lynn. Overall, the collaborative intensity among authors is relatively modest, with most collaborations occurring within small, closely-knit groups.

**Figure 5b** illustrates the co-citation network of authors. Leveraging VOSviewer, authors are categorized into three primary clusters based on the intensity of their co-citation relationships, with each cluster denoted by a distinct color. The red cluster encompasses authors such as Folstein, MF; Pournelle, GH; and Bohannon, RW. The green cluster includes authors like Veronese, N; Guralnik, JM; and Fried, LP. The blue cluster features authors such as Tombaugh, TN; Hausdorff, JM; and Louis, ED. The size of the circles in the diagram corresponds to the co-citation frequency of each author, with larger circles indicating higher co-citation weights. The authors are the most highly cited within their respective clusters.

### 3.5 Distribution of journals

Research on gait-related depression spans across 341 journals. **Table 4** presents the top 10 journals ranked by publication volume and citation frequency. BMC Geriatrics leads with 33 publications, followed by Journals of Gerontology Series A-Biological Sciences and Medical with 30 publications; all other journals published fewer than 30 articles. The most cited journals include Journals of Gerontology Series A-Biological Sciences and Medical with 1570 citations, Journal of the American Medical Directors Association with 1125 citations, and Journal of the American Geriatrics Society with 1091 citations; all other journals collectively received fewer than 1000 citations.

**Table 4:** The top 10 journals by publication volume and citation frequency.

Rank	Journals	Publications	Journals	Co-citations
1	Bmc Geriatrics	33	Journals of Gerontology Series A-Biological Sciences and Medical	1570
2	Journals of Gerontology Series A-Biological Sciences and Medical	30	Journal of the American Medical Directors Association	1125
3	Journal of the American Geriatrics Society	29	Journal of the American Geriatrics Society	1091
4	Plos One	22	Movement Disorders	986
5	International Journal of Environmental Research and Public Health	21	Archives Of Physical Medicine and Rehabilitation	729
6	American Journal of Geriatric Psychiatry	20	Plos One	672
7	Geriatrics & Gerontology International	20	American Journal Of Geriatric Psychiatry	640
8	Journal of the American Medical Directors Association	18	Nature Reviews Neurology	566
9	Archives of Physical Medicine and Rehabilitation	17	Geriatrics & Gerontology International	389
10	Neurorehabilitation	15	Bmc Geriatrics	386

**Figure 6:**
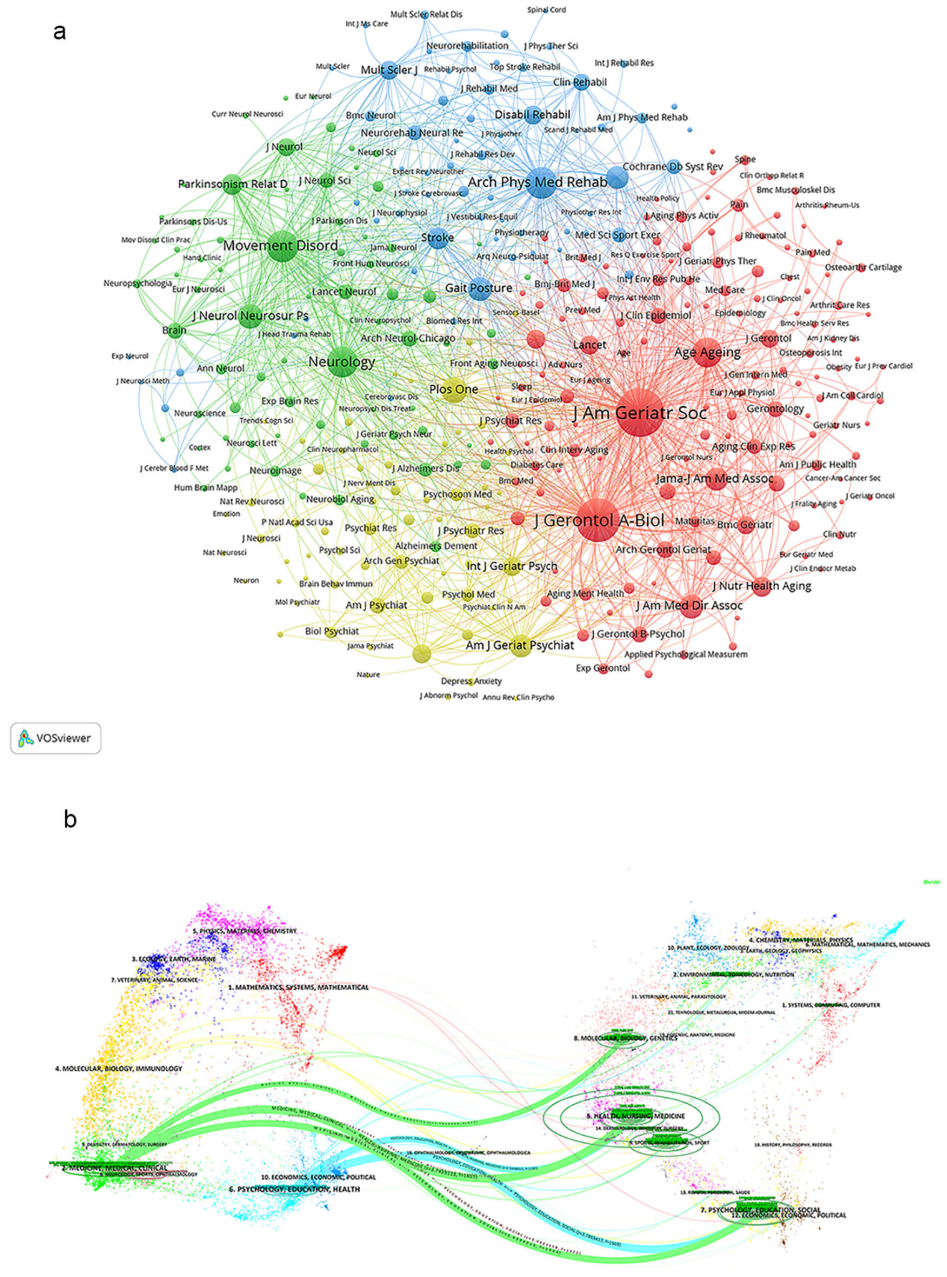
**(A)** Journal citation network. **(B)** Dual -map overlay of journals.

**Figure 6a** illustrates the network of journals publishing articles related to gait-related depression research and their interrelationships. Utilizing VOSviewer, these journals are segmented into four distinct color-coded clusters based on their thematic similarities. The red cluster comprises journals such as J Am Geriatr Soc, J Gerontol A-Biol, and Age Ageing. The green cluster includes Movement Disord, Neurology, and J Neurol Neurosurg Psychiatry. The blue cluster encompasses Arch Phys Med Rehab, Gait Posture, and Stroke. Lastly, the yellow cluster features journals like PLOS ONE, Am J Geriatr Psychiat, and J Affect Disorders.

We utilized knowledge flow analysis to examine the progression of citation and co-citation patterns between citing journals and cited journals (24). The dual-map overlay of journals illustrates the spread of topics, the paths of citations, and the shifts in research hubs among academic journals (24, 25). On the left side of the Dual-map are labels representing citing journals, while on the right side are labels representing cited journals. The colored curves connecting from the citing map to the cited map depict the comprehensive context of each citation. In the citing map, the vertical axis of the ellipse lengthens with an increase in the number of papers published by a journal, while the horizontal axis lengthens with a larger number of authors. The topics covered by Citing Journals predominantly include fields such as MEDICINE, MEDICAL, CLINICAL, MOLECULAR, BIOLOGY, IMMUNOLOGY, NEUROLOGY, SPORTS, and OPHTHALMOLOGY, representing the forefront of research. The topics addressed by Cited Journals mainly encompass areas like MOLECULAR, BIOLOGY, GENETICS, HEALTH, NURSING, MEDICINE, SPORTS, REHABILITATION, PSYCHOLOGY, EDUCATION, SOCIAL, ECONOMICS, POLITICAL, forming the foundational knowledge base **Figure 6b**.

### 3.6 Analysis of keywords

**Figure 7:**
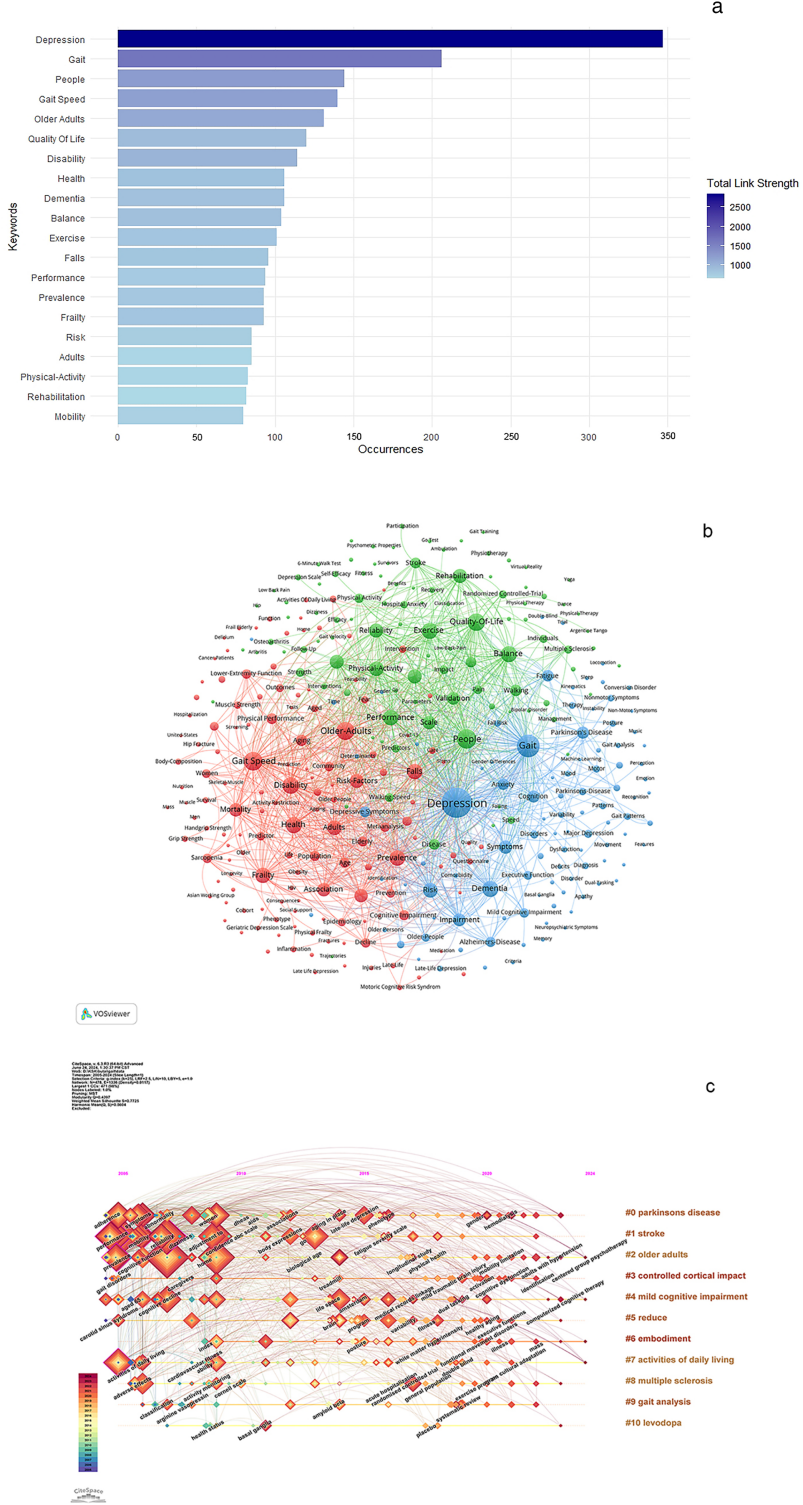
**(A)** Top 20 Keywords. **(B)** Keyword co-occurrence network related to gait in depression. **(C)** Temporal visualization related to gait in depression.

Keywords serve as a fundamental tool to explore the cutting-edge of research on gait-related depression. **Figure 7a** presents the 20 most frequently occurring keywords. The highest frequency is observed with “depression,” followed by “gait,” “people,” “gait speed,” “older adults,” “quality of life,” “disability,” “health,” “dementia,” “balance,” and “exercise.” All other keywords appear fewer than 100 times. **Figure 7b** depicts a keyword co-occurrence network where closely associated keywords are grouped into clusters. These clusters are categorized by different colors. The red clusters, encompassing terms like gait speed, older adults, and disability, primarily pertain to elderly populations and their declining physical capabilities. In contrast, the green clusters, featuring terms such as quality of life, balance, and people, are focused on aspects related to balance and overall quality of life. The blue clusters, including keywords like depression, gait, dementia, and cognition, predominantly relate to cognitive functions. **Figure 7c** illustrates the chronological emergence of these keywords. The size of each square corresponds to the frequency of subsequent occurrences. Keywords listed in the right column represent the thematic clustering observed on the left, predominantly concerning elderly individuals and cognitive decline.

### 3.7 Analysis of reference

**Table 5** presents the top 15 articles ranked by citation frequency, with the most cited being “Executive dysfunction in Parkinson’s disease: A comprehensive review (26),” cited 352 times. This review (26) highlights that executive dysfunction manifests early in Parkinson’s disease (PD), affecting attentional control, planning, and decision-making. Dopaminergic treatment’s impact on executive deficits varies, with implications for motor symptoms like postural instability and gait freezing, alongside psychiatric issues such as depression and apathy, significantly impacting patients’ daily lives. The second most cited article, with 349 citations, is titled “Exercise and Mental Health: Many Reasons to Move (27).” This review (27) scrutinizes the potential of physical exercise as an adjunctive therapy for neuropsychiatric disorders and cognitive decline in aging, emphasizing its capacity to alleviate stress effects and enhance neurotrophic factors, neurotransmitter release, neurogenesis, and cerebral blood flow modulation. Recent studies underscore the substantial influence of exercise on brain health and successful cognitive aging. The article “Cognition, Emotion, and Social Networking in the Community-Dwelling Frail Elderly: A Randomized Clinical Trial” authored by Santabalbina et al. (28), ranks third in citations with 319 mentions. It details a single-blind, interventional, controlled, simple randomized study that examined the impact of a supervised multicomponent exercise program (MEP) on frail older adults. Cognitive assessments, emotional evaluations, and measures of social support demonstrated significant enhancements in the MEP group compared to controls. These results underscore the potential efficacy of structured multicomponent exercise programs in mitigating frailty and improving diverse aspects of health among frail older adults residing in communities. All other articles in the dataset have garnered citations numbering less than 300 each.

**Table 5:** The top 15 articles by citation frequency.

Rank	Article title	Authors	Source title	Year	Citations	DOI	Document Type
1	Executive dysfunction in Parkinson’s disease: A review	Dirnberger, G, et al. (26)	Journal of Neuropsychology	2013	352	10.1111/jnp.12028	Review
2	Exercise and Mental Health: Many Reasons to Move	Deslandes, A, et al. (27)	Neuropsychobiology	2009	349	10.1159/000223730	Review
3	A Multicomponent Exercise Intervention that Reverses Frailty and Improves Cognition, Emotion, and Social Networking in the Community-Dwelling Frail Elderly: A Randomized Clinical Trial	Tarazona-Santabalbina, et al. (28)	Journal of the American Medical Directors Association	2016	319	10.1016/j.jamda.2016.01.019	Article
4	Dual-Task Decrements in Gait: Contributing Factors Among Healthy Older Adults	Hausdorff, JM; Schweiger, A, et al. (29)	Journals of Gerontology Series A-Biological Sciences and Medical Sciences	2008	299	10.1093/gerona/63.12.1335	Article
5	Cerebral small vessel disease: from a focal to a global perspective	ter Telgte, A, et al. (30)	Nature Reviews Neurology	2018	292	10.1038/s41582-018-0014-y	Review
6	The benefits of exercise training in multiple sclerosis	Motl, RW, et al. (31)	Nature Reviews Neurology	2012	274	10.1038/nrneurol.2012.136	Review
7	Embodiment of Sadness and Depression-Gait Patterns Associated with Dysphoric Mood	Michalak, J, et al. (32)	Psychosomatic Medicine	2009	272	10.1097/PSY.0b013e3181a2515c	Article
8	Health-related quality of life in early Parkinson’s disease: The impact of nonmotor symptoms	Duncan, GW, et al. (33)	Movement Disorders	2014	254	10.1002/mds.25664	Article
9	Anxiety Disorders in Parkinson’s Disease: Prevalence and Risk Factors	Dissanayaka, NNW, et al. (34)	Movement Disorders	2010	244	10.1002/mds.22833	Article
10	Long-term morbidities following self-reported mild traumatic brain injury	Vanderploeg, RD, et al. (35)	Journal of Clinical and Experimental Neuropsychology	2007	238	10.1080/13803390600826587	Article
11	The relationship between pain, neuropsychological performance, and physical function in community-dwelling older adults with chronic low back pain	Weiner, DK; Rudy, TE, et al. (36)	Pain Medicine	2006	224	10.1111/j.1526-4637.2006.00091.x	Article
12	Performance-based physical function and future dementia in older people	Wang, L; Larson, EB, et al. (37)	Archives of Internal Medicine	2006	220	10.1001/archinte.166.10.1115	Article
13	Impact of physical frailty on disability in community-dwelling older adults: a prospective cohort study	Makizako, H; Shimada, H, et al. (38)	BMJ Open	2015	184	10.1136/bmjopen-2015-008462	Article
14	Depression and frailty in later life: a synthetic review	Mezuk, B, et al. (39)	International Journal of Geriatric Psychiatry	2012	183	10.1002/gps.2807	Review
15	Balance score and a history of falls in hospital predict recurrent falls in the 6 months following stroke rehabilitation	Mackintosh, SF, et al. (40)	Archives of Physical Medicine and Rehabilitation	2006	180	10.1016/j.apmr.2006.09.004	Article

**Figure 8:**
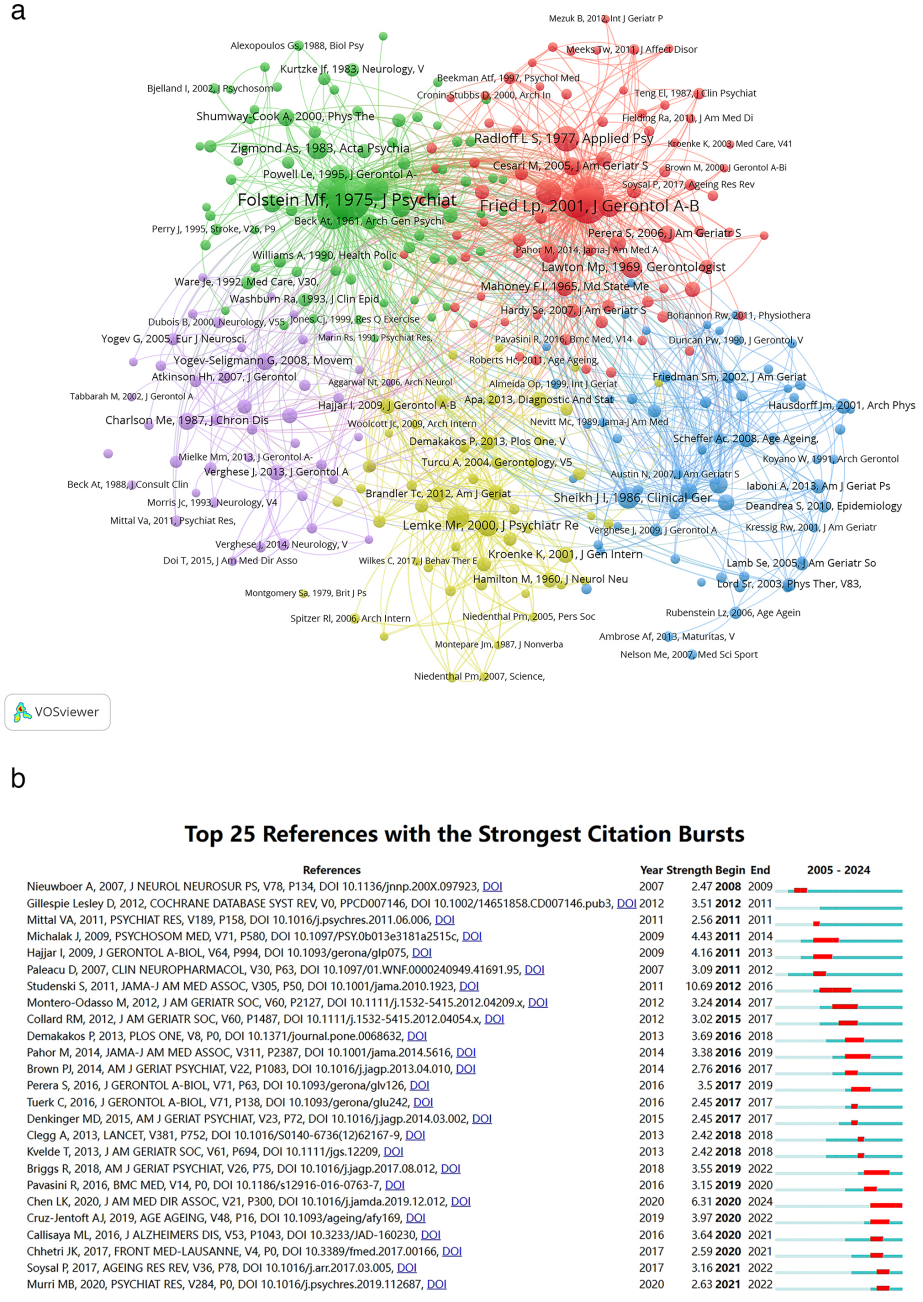
**(A)** Co-citation networks among references; node area is scaled to the frequency of occurrence. **(B)** The 25 references with the highest citation burst strengths are presented.

Reference co-citation analysis, which examines relationships among references based on their shared citation frequency, was employed to analyze reference citation patterns. **Figure 8a** presents a relational diagram depicting these associations among studies, categorized into five main clusters represented by distinct colors. The red cluster includes seminal references such as Fried LP, 2001 (41); Studenski S, 2011 (42); and Guralnik JM, 1994 (43). The green cluster comprises references such as Folstein MF, 1975 (44); Podsiadlo D, 1991 (45); and Yesavage JA, 1983 (46). References in the blue cluster include Tinetti ME, 1990 (47); and Yardley L, 2005 (48); Scheffer Ac, 2008 (49). The yellow cluster encompasses Lemke MR, 2000 (50); Michalak J, 2009 (32); and Hausdorff JM, 2004 (51). Lastly, the purple cluster includes references such as Yogev-Seligmann G, 2008 (52); Montero-Odasso M, 2012 (53); and Verghese J, 2007 (54). In the figure, the size of each circle correlates with its co-citation weight, indicating the prominence of these references within their respective color-coded clusters.

**Figure 8b** illustrates the top 25 references exhibiting the most pronounced citation bursts. The earliest burst was observed in 2008. Titled “Cueing training in the home improves gait-related mobility in Parkinson’s disease: the RESCUE trial” authored by Nieuwboer A et al. (55), this study was published in 2007 in the J NEUROL NEUROSUR PS journal. Studenski S et al.’s paper (42) “Gait speed and survival in older adults” featured in JAMA in 2011, achieved the highest citation burst intensity of 10.69. Notably, 2011 and 2020 emerged as pivotal years with four citation bursts each.

## 4 Discussion

Our bibliometric analysis on gait in depression is executed through the utilization of VOSviewer and CiteSpace. This study spans publications from January 1, 2005, to June 21, 2024, encompassing contributions from 59 countries or regions, 1516 institutions, and 4781 authors. Our analysis revealed that the publication output reached its zenith with 102 papers in 2020, while citation frequency attained its pinnacle at 3920 citations in 2022 (**Figure 2**). In the ensuing years, both publication volume and citation frequency experienced varying degrees of decline.

The analysis of countries or regions reveals that in the field of gait in depression, the USA significantly outpaces other nations in both publication volume and citation frequency, establishing itself as the most influential country in this domain (**Table 1**). Additionally, among the top 10 institutions by publication volume, eight are based in the USA, and among the top 10 institutions by citation frequency, six are also from the USA (**Table 2**). The University of Pittsburgh ranks first in both publication volume with 29 papers and citation frequency with 1204 citations, highlighting its predominant impact in the field of gait in depression. In terms of collaboration, the USA occupies a central position with the highest and strongest connections. Furthermore, countries with higher publication volumes such as Canada, the United Kingdom, and China also maintain close exchanges and collaborations with other nations and regions (**Figure 3**).

Shimada, Hiroyuki holds a prominent position in the field of gait and depression, ranking first in citation frequency and second in publication volume (**Table 3**). Hausdorff, JM et al. (29) published a study in 2008 titled “Dual-Task Decrements in Gait: Contributing Factors Among Healthy Older Adults,” which ranks fourth in citation frequency. Similarly, Makizako, H et al. (38) conducted a prospective cohort study in 2015 titled “Impact of physical frailty on disability in community-dwelling older adults,” which ranks 13th in citation frequency. Both Hausdorff, JM and Makizako, H is notable for their high citation frequencies. Verghese, Joe and Rochester, Lynn, also influential in this field, rank among the top three authors by publication volume alongside Shimada, Hiroyuki, with their citation frequencies also placing in the top ten. This underscores their significant influence and expertise in the field.

We analyzed journals and found that the following 8 journals are not only ranked in the top ten by publication volume but also in citation frequency (**Table 4**): Journals of Gerontology Series A-Biological Sciences And Medical, Journal of The American Geriatrics Society, Archives of Physical Medicine And Rehabilitation, American Journal of Geriatric Psychiatry, Geriatrics & Gerontology International, BMC Geriatrics, Journal of The American Medical Directors Association, and PLOS One. The journals largely focus on topics related to elderly individuals, spanning disciplines such as medicine, biological sciences, geriatric health, rehabilitation, mental health, and neuroscience. This thematic focus corresponds with the conclusions drawn from the dual-map analysis depicted in **Figure 6b**.

Keywords analysis is a critical method in studying depression and gait, offering insights into current research trends and focal points. By examining frequently occurring keywords, researchers can identify core themes and major issues of interest. The keywords in this study—depression, gait, people, gait speed, older adults, quality of life, disability, health, dementia, balance, exercise, falls, performance, prevalence, frailty, risk, adults, physical activity, rehabilitation, mobility—encompass aspects such as depression, aging, cognitive function, and interventions (**Figure 7a**). These keywords reflect the multidimensional and extensive nature of research on depression and gait.

The frequent occurrence of keywords such as ‘depression,’ ‘gait,’ and ‘gait speed’ reflects growing academic interest in the potential associations and clinical implications of gait-related characteristics in the context of depression. Karl E. Friedl et al. (56) propose that mood states are discernible through specific types of human movement, such as slumped posture and shuffling gait, which serve as indicators of depression. Their study found that individuals with major depression showed decreased gait velocity, reduced arm swing, limited vertical head movements, increased lateral swaying of the upper body, and shorter stride length with decreased double limb support compared to individuals who had never experienced depression. In exploratory experiments, Yameng Wang et al. (16) also noted comparable findings. Moreover, college students experiencing sad or happy moods induced by music displayed similar gait characteristics when in a state of sadness (32). Emma Elkjær et al. recently conducted a meta-analysis (57) revealing that individuals with depression exhibit reduced gait speed, step length, stride length, step width, steps taken to turn, and stepping reaction time, alongside increased overall abnormal movement. Additionally, they demonstrate greater instability and a larger center-of-pressure, as well as increased head flexion and reduced joint range of motion in the knees, hips, wrists, shoulders, and elbows. These findings likely strengthen the argument for a distinct gait pattern associated with depression. However, present findings do not definitively substantiate that gait patterns are exclusive symptoms of depression (56). Current studies typically investigate depression as a homogeneous entity. Patients with depression may manifest either psychomotor retardation or psychomotor agitation, introducing potential inconsistencies in results. Future research should emphasize investigating the temporal and spatial relationships between these unique gait patterns and the disorder, as well as establishing criteria for defining these patterns as distinctly associated with the disorder.

Gait characteristics associated with depression may vary by age, gender, and occupation. Recent studies have indicated that adult patients with depression often exhibit significant impairments in posture, balance, and gait functions, while gait abnormalities in older adults with depression may result from the interplay of physical illness, cognitive impairment, and emotional disturbances (58). Other research has demonstrated that daily gait patterns can provide additional information for predicting the severity of depressive symptoms (59). Age is a key moderator of depression-related gait features. Studies have shown that gait abnormalities are more pronounced in older patients with depression, particularly characterized by slower gait speed and increased gait variability. These changes may be closely related to age-associated neurodegenerative processes and cognitive decline (58). Gender differences also warrant attention. Evidence suggests that in healthy populations, males and females exhibit distinct gait parameters—males generally present with longer stride length and faster gait speed, while females tend to show higher step frequency and different patterns of gait symmetry. However, there is currently a lack of systematic research on gender-related gait differences among patients with depression, highlighting the need for further investigation. Occupational factors may also influence gait performance in individuals with depression through psychosocial stress mechanisms. A systematic review (60) has reported that adverse occupational psychosocial stress is significantly associated with impaired autonomic nervous system function, as indicated by reduced heart rate variability, suggesting that prolonged stress may disrupt parasympathetic activity and autonomic balance. Additionally, another study (61) found that occupational role stress—such as role conflict and ambiguity—elicited stronger acute cortisol responses (p = 0.016), indicating that high-stress work may activate the hypothalamic-pituitary-adrenal (HPA) axis, potentially impairing gait stability and motor control.

Despite the common assumption that motor disturbances are more pronounced in older individuals (62), the moderation analysis indicated no significant moderating effects of age (57). Across various age groups, individuals face challenges such as reduced gait speed, step length, stride length, and step width, as well as increased overall abnormal movement. These issues not only restrict their daily activities but also increase the risks of falls and injuries, impacting their health and quality of life. Such challenges may contribute to conditions of frailty and disability, further limiting their daily activities and social participation.

In depression, concurrent with cognitive decline including reductions in attention and memory is evident. Additionally, depressive symptoms represent a risk factor for mild cognitive impairment in older adults (63). Major depression correlates with increased variability in stride time, stride velocity, and swing time during dual-task activities, indicating compromised executive function and visual memory (64). Depressed elderly patients exhibit slower walking speed, shorter strides, reduced minimum toe clearance, and greater variability in stride length compared to healthy controls (65). Additionally, dual-task conditions exacerbate these impairments, resulting in further reductions in gait speed, stride length, and prolonged swing time relative to single-task conditions (65). In 2021, Mehmet Ilkin Naharci et al. (66) found that slower gait speed is independently associated with depressive symptoms in older adults with mild cognitive impairment. Depression and cognitive impairment may present with distinct gait characteristics, both of which affect stability and balance, thereby increasing the risk of falls. Future research needs to further explore whether depression and cognitive impairment exhibit distinct gait features and elucidate the differences between them.

The high fall rates in depression may be explained by abnormal posture. Gait abnormalities increase the risk of falls, severely affecting patients’ quality of life and mobility. Proactive intervention targeting abnormal gait is crucial for reducing fall risk and enhancing quality of life in individuals with depression. For these patients, altering emotional states through short bodily manipulations has been found to be effective (57), consistent with recent studies in healthy populations (67–69). The major motor system may be a direct target for interventions in depression (57). The number of studies related to physical inactivity has increased significantly (18). Reduced physical activity due to depression can be mitigated through exercise, which is effective in reducing depressive symptoms with just moderate to vigorous physical activity replacing 60 minutes of sedentary time (70–72). Additionally, there is evidence suggesting that (32, 73) certain therapeutic approaches, including mindfulness, can influence the motor system.

With the continuous advancement of technology, artificial intelligence (AI) and wearable devices have gradually become essential tools in gait research related to depression. Bibliometric analysis reveals a sustained growth trend over the past two decades in studies involving wearable health sensors, with an average annual growth rate of approximately 20.7%, providing more ecologically valid and scalable methods for long-term dynamic gait monitoring in depression (74). Additionally, Azliyana Azizan et al. (75) conducted a bibliometric review of sensor-based fall prevention research in the elderly, identifying key research hotspots and future opportunities, which reflects the broad potential of sensor technologies in health monitoring applications.

From the perspective of algorithm development, the introduction of AI models has significantly advanced the intelligence of gait recognition. Nan Zhao et al. (76) utilized Kinect to capture gait images combined with machine learning approaches to accurately predict depression levels, demonstrating the feasibility of integrating visual perception and machine learning. Haifeng Lu et al. (77) proposed an integrated evaluation framework based on multimedia gait data by applying frequency domain analysis of kinetic and potential energy, markedly improving the objectivity and practicality of depression risk detection among graduate students. Tao Wang et al. (78) developed a gait assessment framework that fuses temporal, frequency, and spatial geometric features, achieving efficient, non-contact depression detection with a classification accuracy of 93.75%, indicating strong effectiveness and robustness. Furthermore, Jingjing Yang et al. (79) enhanced depression gait detection models by applying skeletal data augmentation techniques that preserve key skeletal attributes, resulting in accuracy improvements up to 92.15%.

Overall, current bibliometric trends indicate that the integration of AI and wearable technologies is driving depression gait research from simple feature identification toward more precise risk prediction. This evolving direction is expected to facilitate high-throughput, high-accuracy, and highly adaptable clinical applications, representing a major research focus and future trend in the field.

This study has several considerations to note. First, the decline in publications after 2020 may be related to incomplete indexing of recent literature, which could affect the completeness of the dataset. Second, data were obtained solely from the Web of Science Core Collection; incorporating additional databases such as Scopus and PubMed could provide a broader scope of relevant literature and enrich data diversity. Furthermore, bibliometric analysis primarily offers a macroscopic view of research trends and structures and does not directly assess clinical efficacy or causality. Therefore, the findings should be viewed as hypothesis-generating and warrant further validation through clinical studies. Additionally, characteristics inherent to citation practices, variability in keyword definitions, and the absence of individual-level data are factors to consider when interpreting the results.

Future research may benefit from integrating bibliometric methods with qualitative content analysis or systematic clinical reviews to deepen the understanding of gait alterations in depression. The application of machine learning–enhanced text mining, full-text semantic analysis, and multi-database triangulation can further improve the comprehensiveness and granularity of future studies. Natural language processing (NLP) shows promising potential in identifying emerging psychosomatic gait phenotypes across interdisciplinary medical and psychiatric databases. Moreover, fostering interdisciplinary collaboration among psychiatry, biomechanics, computer science, and geriatrics is crucial to translating bibliometric insights into clinical practice, particularly in areas such as digital gait assessment and AI-assisted mental health screening.

## 5 Conclusions

In our study, we employed bibliometric analysis to examine the research progress and future trends of gait in depression over the past 20 years. The number of publications and citations related to gait in depression has been increasing annually, with a slight decline observed in recent years. The USA holds a central position in this field. The University of Pittsburgh, the University of California, San Francisco, and Harvard Medical School are prominent institutions within this domain. Influential authors include Joe Verghese, Hiroyuki Shimada, and Lynn Rochester. Current research frontiers encompass the identification of distinctive gait patterns in depression, gait characteristics in the elderly, and the association between gait and cognitive decline. Additionally, there are exploratory studies investigating potential interventions for abnormal gait in depressive populations. This bibliometric analysis highlights these research trends and frontiers, providing valuable references for scholars interested in significant topics and potential collaborations. It is important to emphasize that most existing studies are observational and exploratory in nature, lacking systematic clinical validation. Therefore, the findings of this study should be considered hypothesis-generating rather than confirmatory. Future research is needed to further elucidate the mechanisms underlying gait changes in depression and to evaluate the potential clinical utility of related interventions.

## Data Availability

The original contributions presented in the study are included in the article/supplementary material, further inquiries can be directed to the corresponding author/s.
